# Localization and Expression of Zonula Occludins-1 in the Rabbit Corneal Epithelium following Exposure to Benzalkonium Chloride

**DOI:** 10.1371/journal.pone.0040893

**Published:** 2012-07-18

**Authors:** Wensheng Chen, Jiaoyue Hu, Zhenhao Zhang, Lelei Chen, Hui Xie, Nuo Dong, Yongxiong Chen, Zuguo Liu

**Affiliations:** Eye Institute and affiliated Xiamen Eye Center of Xiamen University, Fujian Provincial Key Laboratory of Ophthalmology and Vision Science, Fujian, China; Northwestern University Feinberg School of Medicine, United States of America

## Abstract

Preservatives are a major component of the ophthalmic preparations in multi-dose bottles. The purpose of this study was to investigate the acute effect of benzalkonium chloride (BAC), a common preservative used in ophthalmic preparations, on the localization and expression of zonula occludens (ZO)-1 in the rabbit corneal epithelium *in vivo*. BAC at 0.005%, 0.01%, or 0.02% was topically applied to one eye each of albino rabbits at 5 min intervals for a total of 3 times. The contralateral untreated eyes served as controls. The following clinical indications were evaluated: Schirmer test, tear break-up time (BUT), fluorescein and rose Bengal staining. The structure of central cornea was examined by *in vivo* confocal microscopy, and the corneal barrier function was evaluated by measurement of corneal transepithelial electrical resistance and permeability to carboxy fluorescein. Whole mount corneas were analyzed by using fluorescence confocal microscopy for the presence of ZO-1, 2, occludin, claudin-1, Ki67 and cell apoptosis in the epithelium. The expression of ZO-1 in the corneal epithelium was also examined by western blot and reverse transcription-polymerase chain reaction analyses. Exposure to BAC resulted in higher rose Bengal staining scores while no significant changes in BUT, Schirmer and corneal florescein scores. It also induced corneal epithelial cell damage, dispersion of ZO-1 and ZO-2 from their normal locus at the superficial layer and disruption of epithelial barrier function. However, the amounts of ZO-1 mRNA and protein in the corneal epithelium were not affected by BAC treatment. Exposure to BAC can quickly impair the corneal epithelium without tear deficiency. BAC disrupts the tight junctions of corneal epithelium between superficial cells in the rabbit corneal epithelium *in vivo*.

## Introduction

Corneal epithelium forms a barrier to protect the eye from the outside environment and helps to maintain corneal transparency [1]. Tight junctions are the most apical intercellular junctions, and play an important role in the establishment and maintenance of the barrier function [2]–[5]. The tight junctions are composed of different types of membrane proteins such as claudin, occludin and junctional adhesion molecules, membrane-associated proteins such as zonula occludins (ZO)-1, ZO-2, and ZO-3, and actin filaments [6], [7]. ZO-1 is a 220-kDa cytoplasmic protein, which was first described as a component of tight junctions of epithelial and endothelial cells [8]–[12]. It is a member of the membrane-associated guanylate kinase (MAGUK) family of proteins characterized by protein-protein interaction domains (PZD domains), Src homology domain (SH3), and the guanylate kinase (GUK) domain [7]. In addition to maintaining barrier function in epithelia, ZO-1 has been found to function as a cell signaling protein for multiple cell types, contributing to the regulation of cell proliferation and differentiation [13]. In the corneal epithelium, ZO-1 is expressed in superficial cell layer and has been considered a marker of the tight junction [14]–[16]. Disruption of corneal epithelial barrier function results in ocular irritation [17], [18], and increased risk for microbial infections [19], [20].

Ophthalmic preparation is used not only to treat ocular surface disorders but also for intraocular diseases such as glaucoma. Preservatives are a major component of the ophthalmic preparations, providing antimicrobial activity and preventing decomposition of the active drug in multi-dose bottle. As a soluble antimicrobial agent and surfactant, benzalkonium chloride (BAC) is the most commonly used preservative in ophthalmic solutions for its apparently good safety/efficacy profile [21]. However, many studies have revealed that long-term use of topical drug with BAC may induce ocular discomfort, tear film instability, loss of goblet cells, inflammation, conjunctival squamous metaplasia, epithelial cell apoptosis, subconjunctival fibrosis and even the potential risk of failure for further glaucoma surgery [21].

BAC is most often used at a concentration of 0.01% (ranging from 0.004% to 0.02%) in ophthalmic preparations [22]. In 1944, Swan found that BAC causes punctuate corneal epithelial damage at concentration of 0.04%, and edema and cellular desquamation with corneal epithelial lesions at a concentration of 0.1% [23]. Pauly et al. have shown that topical application of high concentration of BAC (0.25% or 0.5%) lead to corneal endothelial cell edema, even disappearance [24]. Recent studies have proven that exposure to BAC induces a continuous decline in corneal transepithelial electric resistance (TER) in rabbit, suggestive of the disruption of tight junction [25], [26]. However, *in vivo* effect of BAC on the localization and expression of ZO-1, the marker of tight junction, in the corneal epithelium remains largely unclear.

In the present study, we investigated the acute effect of BAC on the localization and expression of ZO-1 in the rabbit corneal epithelium *in vivo*. We found that exposure to BAC can quickly disrupt the tight junctions between superficial cells in the rabbit corneal epithelium. The significance of the findings is further discussed.

## Methods

### Antibodies and Reagent

BAC was purchased from Sigma (St. Louis, MO); pentobarbital sodium from Abbott Laboratories (North Chicago, IL); mouse anti-rabbit ZO-1 antibody, mouse anti-rabbit ZO-2 antibody, mouse anti-rabbit claudin-1 and mouse anti-rabbit occludin from Zymed (Carlsbad, CA); rat monoclonal antibodies for Ki67 from DakoCytomation (MIB-1; Glostrup, Denmark); TUNEL apoptosis detection kit was purchased from Roche Diagnostics (Meylen, France); Alexa488-conjugated goat anti-mouse IgG, goat anti-mouse IgG and Alexa594-conjuagated mouse anti-rat IgG from Molecular Probes (Eugene, OR); 4,6-diamidino-2-phenylindole (DAPI) and bovine serum albumin (BSA) from Vector Laboratories (Burlingame CA); Dulbecco modified Eagle medium (DMEM) and Dispase II from Invitrogen Corp (Carlsbad, CA); mouse anti-rabbit β actin antibody from Sigma; Horseradish peroxidase-conjugated goat antibody to mouse immunoglobulin G (IgG), nitrocellulose membranes and an enhanced chemiluminescence (ECL) kit from GE Healthcare UK (Chalfont, UK).

### Animals and BAC Treatment

A total of 36 male white New Zealand rabbits (purchased from Shanghai Medical Laboratory Animal Center, Shanghai, China) weighing between 1.5 and 2.0 kg were randomly assigned to three groups of 12 rabbits each. The rabbits were quarantined and acclimatized a week before the experiments in the Eye Institute of Xiamen University, Xiamen, China. The animals were housed individually in cases at constant room temperature (19–23°C) and humidity of 40–50% with a constant 12-hour light-dark cycle. They were fed with chow and water ad libitum. The animals were screened for ocular surface disease with a handheld biomicroscope before experimental procedure and were excluded if any disease was found. BAC at 0.005%, 0.01%, or 0.02% was applied to one eye of rabbit, with the second eye of each animal serving as a BAC-free control. Procedures involving experimental animals were performed in accordance with ARVO Statement for Use of Animals in Ophthalmic and Vision Research and approved by the animal ethics committee of Xiamen University School of Medicine (approval ID: XMUMC 2011-10-08).

### Clinical Ocular Surface Evaluations

Each rabbit underwent a clinical ocular surface examination, including Schirmer’s test, Fluorescein and Rose Bengal staining, and tear break-up time (BUT).

Schirmer’s test: Schirmer paper strips (Tianjin Jingming New Technology Development Co., Ltd., Tianjin China) was inserted into the lower mediolateral third of the conjunctival fornix of eyes for 5 minutes without anesthesia. After the strip was removed, the mount of wetting in millimeter was recorded to an accuracy of 0.5 mm. The test was repeated three times, and then the mean value was obtained.

BUT and Corneal Fluorescein Staining: Two µl of 1% sodium fluorescein was instilled into the lower fonix of the conjucntiva. The rabbit was allowed to blink several times to distribute the fluorescein evenly on the cornea. The time from opening of the eyes to the appearance of the first dry spot in the central cornea was measured 3 times and the mean was recorded with a cobalt blue filter under a slit-lamp microscope (BQ900® Haag-Streit, Bern, Switzerland). Two minutes later, corneal fluorescein staining intensity was also examined and graded under the slit-lamp microscope.

Rose bengal staining: After topical application of proparacaine (Alcaine; Alcon, Fort Worth, TX), 1 drop of solution of 1% rose bengal was applied to the eyes, and the excess rose bengal was washed out with saline. The ocular surface was examined and graded under the slit lamp microscope [27].

### *In Vivo* Confocal Microscopy

After clinical in rabbits anesthetized with xylazine (1 mg/kg body weight; Bayer, Shawnee Mission, KS) and sodium pentobarbital (20 mg/kg, Abbott Laboratories, North Chicago, IL). The Heidelberg Retina Tomograph III/Rostock Cornea Module (Heidelberg Engineer GmbH, Heidelberg, Germany) laser scanning *in vivo* confocal microscopy was used to examine the structure of the central cornea. It used a 60× water-immersion objective lens (Olympus, Hamburg, Germany) and a 670-nm diode laser as a light source with a wavelength of 670-nm. Images consist of 384×384 pixels, allowing a scanning area of 400 µm^2^ with lateral and vertical resolutions of both 1 µm and a magnification up to 800 times. Before examination, a drop of carbomer gel (Alcon Laboratories, Fort Worth, TX) was applied as coupling medium between the applanating lens and the cornea. The center of the cap was applanated onto the central cornea by adjusting the controller, and in vivo digital images of the cornea were visualized directly on the computer screen. The central cornea was examined and more than 10 images were taken for each of the following structure: superficial and basal epithelium, stroma, and endothelium. The mean central corneal thickness was calculated based on the depth difference between the most superficial epithelium and the endothelium and was recorded as the average of a minimum of three individual acquisitions. All measurements were performed by a single investigator masked to the specific experimental conditions. At the end, the superficial epithelial cell size and the central corneal thickness were calculated. Cell density was recorded as cells per square millimeter. Based on 10 images, the means and standard deviations were calculated for each parameter.

### Measurements of Corneal TER

Corneal TER was measured as previously described [25]. Briefly, a 1.0-mm-diameter Ag/AgCl electrode (Physiotech, Tokyo, Japan) was inserted into the anterior chamber through a small incision in the peripheral cornea, which had been made with an 18-gauge sharp needle (Terumo, Tokyo, Japan). Using biomedical adhesive (Alon-Alpha A; Sankyo), a 6.0-mm-internal diameter (0.28-cm^2^ inner area) nitrile rubber O-ring (Union Packing; SAN-EI, Osaka, Japan) was fixed on the cornea. Then, 60 µL of HBSS was placed inside the ring at the center of the cornea, and the other electrode was carefully placed on the cornea. The TER was measured using a volt-ohm meter (EVOMX; World Precision Instruments, Sarasota, FL), which generates ±20-µA alternating current (AC) square wave current at 12.5 Hz, and data were recorded using a thermal arraycorder (WR 300-8; Graphtech, Tokyo, Japan).

### Measurement of Permeability to Carboxy Fluorescein (CF)

Corneal epithelial barrier function was also evaluated on the basis of measurement of corneal permeability to carboxy fluorescein (CF; 0.3%, Cohasset, MA) [28]. Briefly, 10 minutes after 40 µL of CF was applied to each ocular surface, the animals (n = 3 per group) were euthanized with overdose of pentobarbital sodium and corneas were excised. Each cornea was washed 3 times in 1 mL of balanced salt solution (BBS; Alcon Laboratories, Fort Worth, TX) for 5 minutes per wash, cut into 4 pieces and places in tube with 1 mL of BSS. Each tube was wrapped in aluminum foil to protect the solution from light and placed on an orbital shaker for 90 minutes. The concentration of CF (nmol/µL) was measured by using a Gilford Fluoro IV fluorometer (Corning, Oberlin, OH).

### Immunofluoresecence Microscopy

After *in vivo* examinations, rabbits were killed with a lethal dose of pentobarbital sodium (100 mg/kg) injected intravenously. After the eyes had been enucleated and fixed in PBS with 4% paraformaldehyde for 3 minutes, under a dissecting microscope (Model SZ40; Olympus, Tokyo, Japan), the retina, lens and iris were discarded, and four incisions were made in each cornea. Subsequently, the corneas were permeabilized with acetone for 3 minutes at −20°C. After washing in PBS with 1% Triton X-100 and 1% dimethyl sulfoxide (DMSO; TD buffer), the tissue blocks were incubated in 1% BSA diluted in TD buffer for 1 hour to block nonspecific binding. Then, the tissues were incubated overnight at 4°C with antibodies to ZO-1, ZO-2, occludin or claudin-1. The next day, they were washed in TD buffer, placed in Alexa Fluor 488-conjugated secondary antibody for 1 hour at 4°C. Afterward, the tissues were incubated in anti-Ki67 antibody for 8 h with agitation. The tissues were then rinsed with TD buffer and placed in placed in Alexa Fluor 594-conjugated secondary antibody for 2 h with agitation. After distilled water wash, the whole-mount cornea tissues were mounted epithelial side up on a slide and stained with a nuclear fluorescence dye, DAPI. Tissues without primary antibody were used as negative control. All antibodies were optimally diluted in TD buffer. Negative controls included incubation of tissues with preimmune mouse serum instead of primary or secondary antibodies alone.

To measure end-stage apoptosis, in situ TUNEL labeling was performed using the TUNEL apoptosis detection kit. After being rinsed with TD buffer, equilibration buffer was applied to corneal tissues for 45 min. The tissues were further incubated with TdT reaction mix for 2 h, and then immersed in standard saline citrate to stop reaction. After distilled water wash, the whole-mount cornea tissues were mounted epithelial side up a slide and stained with a nuclear florescence dye, DAPI.

Finally, the corneal tissues were analyzed under a laser confocal microscope (Olympus Fluoview 1000; Olympus, Japan). Multiple Z-sections were generated, and only positively labeled cells in epithelium were counted. Using grid, the number of cells with different marker was counted. All the corneal tissues that had been observed on whole mount were cut into sections. These sections were also examined by confocal microscope.

### Western Blot Analysis

Excised corneas were washed several times with PBS, and the endothelial layer was removed mechanically. Proteins of the remaining tissues from each group were extracted with cold RIPA buffer. Equal amounts of proteins of the cell lysates were subjected to electrophoresis on 8% or 11% SDS-PAGE and then electrophorectially transferred to PVDF membrane. After block in 2% BSA for 1 h, the membranes were incubated with primary antibodies for ZO-1 (1∶1000), and β actin (1∶10000) as a loading control overnight at 4°C with gentle rocking. The second day, after three washes with Tris-buffered saline with 0.05% Tween-20 for 10 minutes each, the membranes were incubated with HRP-conjugated goat anti-mouse IgG (1∶10000) for 1 h. Immune complexes were detected with ECL reagent. Band intensities were measured by image analysis software, and those for ZO-1 were normalized by the corresponding value for β actin (developed by Wayne Rasband, National Institutes of Health, Bethesda, MD; available at http:rsb.info.nih.gov/ij/index.html).

### RT-PCR Analysis

Corneal epithelial sheets were harvested as described above, and total RNA was extracted from the sheets using Trizol (Invitrogen, Carlsbad, CA) according to the manufacturer’s instructions. The RNA were subjected to the RT-PCR analysis (Revert Aid™ First strand cDNA synthesis kit, Fermentas EU) based on the Taq DNA polymerase, dNTP and reaction buffer (Takara, China). The PCR protocol was designed to maintain amplification in the exponential phase. Sequence of the PCR primers were as follows: ZO-1 sense, 5′-GTCTGCCATTACACGGTCCT-3′; ZO-1 antisense, 5′-GGTCTCTGCTGGCTTGTTC-3′; glyceraldehyde-3-phosphate dehydrogenase (G3PDH; internal control) sense, 5′-ACCACAGTCCACGCCATCAC-3′; and G3PDH antisense, 5′-TCCACCACCCTGTTGCTGTA-3′. RT and PCR incubation were performed with a PCR system (GeneAmp 2400-R; Perkin-Elmer, Foster City, CA). RT was performed at 25°C for 5 minutes, 42°C for 3 h, 70°C for 5 minutes, finally cooled to 4°C. PCR were performed at 94°C for 3 minutes, 94°C for 30 seconds, 55°C for 30 seconds, 68°C for 50 seconds, 68°C for 7 minutes. The reaction mixture was finally cooled to 4°C, and the products of amplification were fractionated by electrophoresis on a 2% agarose gel and stained with ethidium bromide. Band intensities were measured by image analysis software (developed by Wayne Rasband, National Institutes of Health, Bethesda, MD; available at http:rsb.info.nih.gov/ij/index.html) and those ZO-1 were normalized by the corresponding value for G3PDH.

### Statistical Analysis

Quantitative data are expressed as mean ± SD from three independent experiments and were analyzed by Dunnett multiple comparison test. P<0.05 was considered statistically significant.

## Results

### Clinical Observations

**Figure 1:**
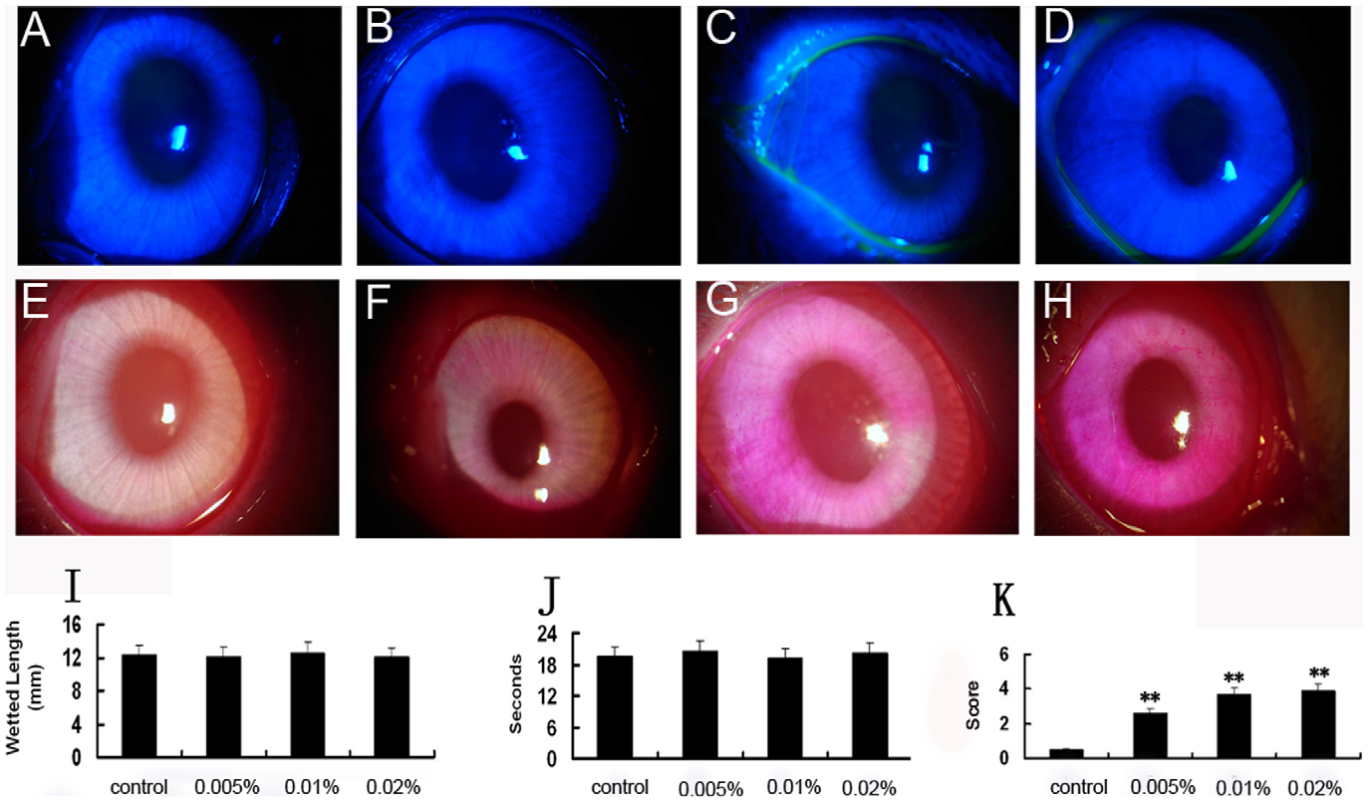
Clinical evaluation of acute effect of BAC on the ocular surface. (A–D) Representative images of corneal fluorescein staining showing no substantial fluorescein staining was apparent in both control (A) and BAC treated eyes (C–D). (E–H) Representative images of corneal rose bengal staining showing the patches and diffuse corneal staining in BAC-treated eyes compared with no obvious staining in the untreated control eye. (I)Schirmer test. (J) BUT. (K) Rose bengal staining score. There were no statistically significant differences between the BAC-treated and control groups in aqueous tear production and BUT. However, rose bengal staining showing statistically significant differences between the BAC-treated and control groups. ** P<0.01 (Dunnett test).

There were no significant differences between control and BAC-treated eyes in aqueous tear production, corneal fluorescein scores and BUT. However, significant increases in the rose bengal scores were observed after BAC treatment (Fig. 1).

### *In vivo* Confocal Microscopy Analysis

**Figure 2:**
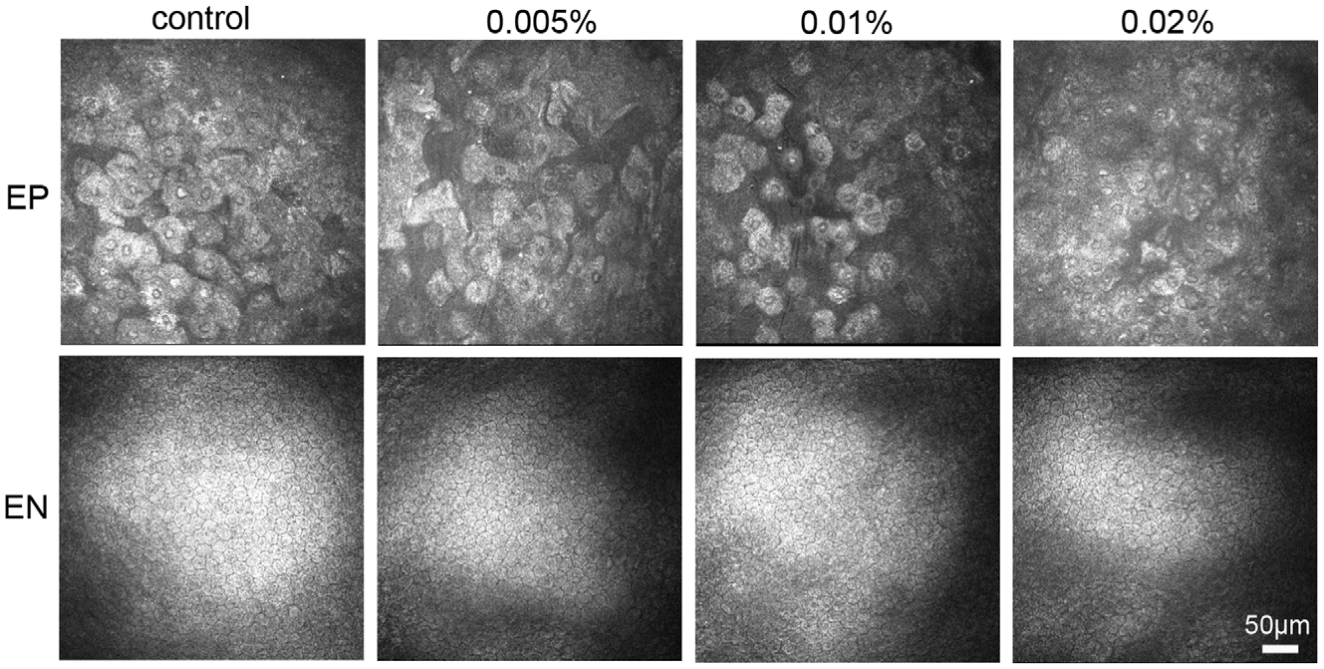
Acute effect of BAC on the morphology of rabbit corneal epithelial superficial and endothelial cells. Note that surface cells in the corneal epithelium of eyes treated with BAC was smaller, with blurry boundaries and abnormal reflectivity nuclei, compared with those of control eye. In contrast, the morphology of corneal endothelial cells did not appear to differ between BAC treated and control eyes. EP: epithelium, EN: endothelium.

**Figure 3:**
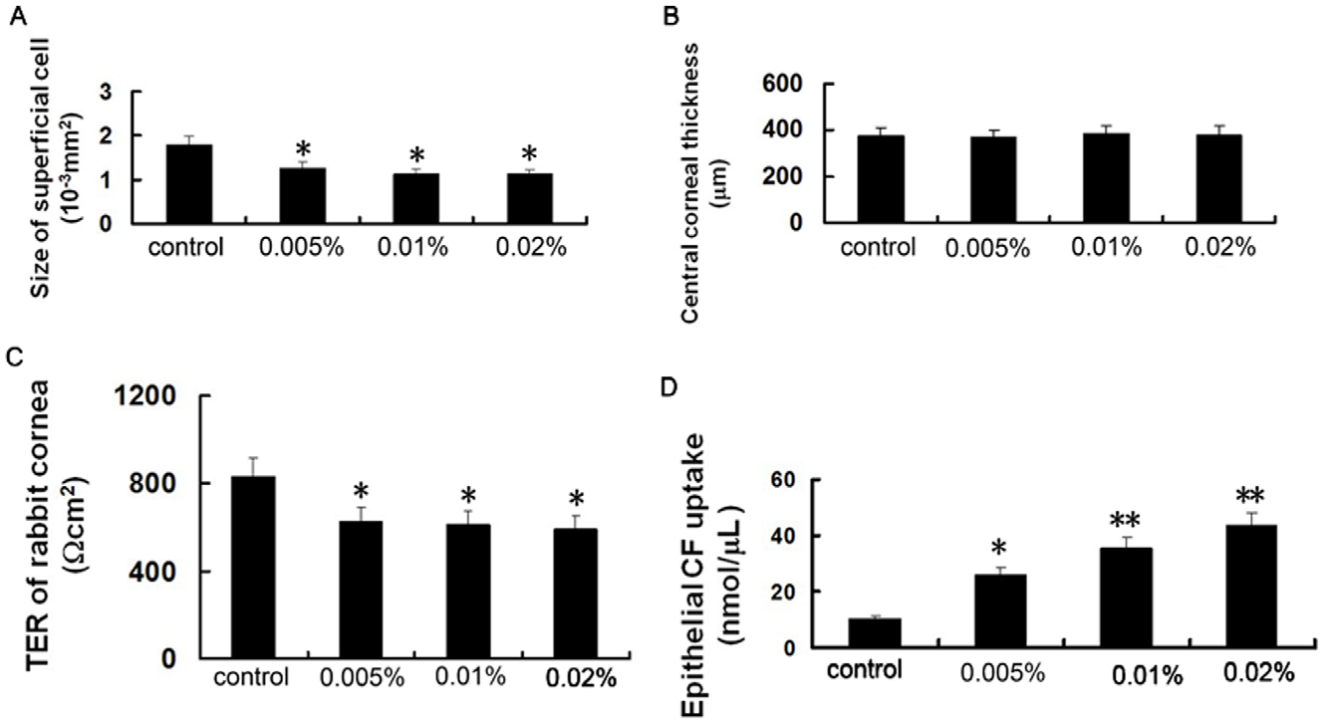
Acute effect of BAC on the size of surface cells in the corneal epithelium, central corneal thickness, corneal TER and CF uptake. Note that the size of surface cells in the corneal epithelium of eyes treated with high concentrations of BAC was significantly smaller than that of control (A), corneal TER was significantly decreased (C) and corneal CF uptake was significantly increased (D) in all BAC treated cornea. In contrast, there was no significant difference between BAC treated and control eyes in central corneal thickness (B). Data show mean ± SD of values from six eyes per group. * P<0.05, ** P<0.01 (Dunnett test).

An *in vivo* confocal microscopy was used to examine the central cornea of rabbits after BAC treatment. The normal corneal superficial epithelium exhibited large, polygonal, squamous cells with brightly reflective nuclei (Fig. 2). In contrast, 0.005% BAC-treated rabbits presented abnormal aspects of the corneal superficial epithelium, with blurry boundaries and hyporeflective nuclei (Fig. 2). Loss of cell boundaries and small hyperreflective nuclei were observed in the eyes treated with 0.01% and 0.02% BAC (Fig. 2). The sizes of the corneal superficial epithelial cells of eyes treated with 0.01% and 0.02% BAC were significantly smaller, by 29.2% (*P*<0.05), 36.5% (*P*<0.05) and 37.8% (*P*<0.05), than that of control eyes, respectively (Fig. 3A). These findings suggest that topical application of BAC can quickly cause damage to the surface cell of the corneal epithelium.

In contrast to these effects of BAC on the corneal superficial epithelium, the morphology of corneal epithelial basal layer, stroma and endothelium did not appear obvious differences between control and BAC treated eyes (Fig. 2). In addition, there was no a significant difference between the control and BAC-treated eyes in central corneal thickness (Fig. 3B).

### Acute Effect of BAC on the Barrier Function of Corneal Epithelium

To assess the acute effect of BAC on corneal barrier function, we measured the corneal TER of living rabbits. The mean TER of normal control rabbit cornea was 832.1±107.3 Ω cm^2^. Exposure of the cornea to BAC resulted in a concentration-dependent decrease in TER, with this effect being significant at the lowest concentration used here (Fig. 3C). These data thus suggested that exposure to BAC can quickly lead to disruption of the barrier function of cornea.

As shown in Figure 3D, treatment with BAC significantly increases epithelial permeability, compared with untreated control.

### Acute Effect of BAC on the Localization of Tight Junction Proteins in the Corneal Epithelium

**Figure 4:**
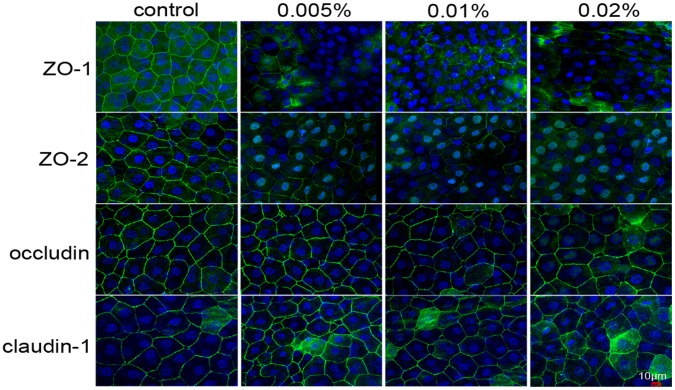
Acute effects of BAC on distribution of ZO-1, ZO-2 occludin and claudin-1 in the rabbit corneal superficial epithelium. Corneal tissue blocks prepared from a control eye or from eyes treated with 0.005%, 0.01% or 0.02% BAC. ZO-1, ZO-2, occludin and claudin-1 staining was observed as a continuous linear pattern along with superficial cell-cell borders in normal rabbit corneal epithelial cells. ZO-1and ZO-2 staining was patchy and discontinuous in the eyes treated with BAC. In contrast, the pattern of occludin and claudin-1distribution in the eyes treated with BAC is similar to that of untreated cells. Data are representative of three independent experiments.

To investigate the acute effect of BAC on the components of tight junction-associated proteins, we examined the distributions of the tight junction proteins ZO-1, ZO-2, occludin and claudin-1 by immunofluorescence microscopy. In untreated corneal epithelia, ZO-1, ZO-2, occludin and claudin-1 were all localized contiguously at the superficial cell-cell boundaries and accordingly stained uniformly at the cell borders (Fig. 4A). To further assess the distribution of TJ-associated proteins in the rabbit corneal epithelium, cryosections were prepared from whole mount corneal tissues whose en face profile had been examined previously [29]. The results revealed that ZO-1, ZO-2 and claudin-1 were all exclusively located in the corneal superficial epithelial layer (data not shown). BAC treatment resulted in a loss of ZO-1and ZO-2 immunoreactivity from the superficial epithelial cellular border, but it did not affect the distribution of occludin and claudin-1 (Fig. 4B–D). These observations thus suggested that exposure to BAC quickly disrupted the localization of ZO-1 and ZO-2 in the rabbit corneal epithelium *in vivo*.

### Acute Effect of BAC on the Expression of ZO-1 Protein and mRNA

**Figure 5:**
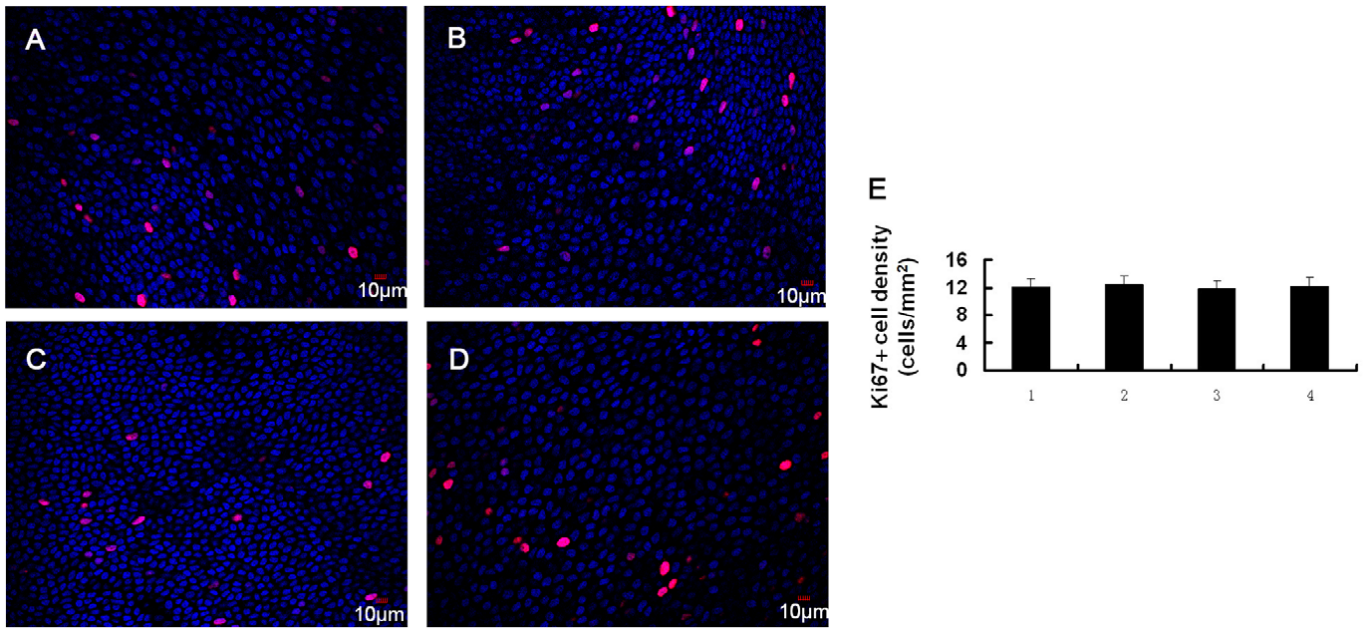
Acute effect of BAC on expression of Ki67 in the rabbit corneal epithelial basal layer. Corneal tissue blocks prepared from a control eye (A) or from eyes treated with 0.005% (B), 0.01% (C) or 0.02% BAC (D). Mean basal Ki 67+ cells were shown in (E). Data show mean ± SD of values from three eyes per group.

The expression of ZO-1 protein and mRNA in the rabbit corneal epithelium was evaluated by western blot analysis and RT-PCR analyses, respectively. The results showed that short exposure to BAC had no significant effect on the amounts of ZO-1 protein and mRNA in the corneal epithelium (Fig. 5).

### Acute Effect of BAC on Corneal Epithelial Cell Proliferation and Apoptosis

To further investigate the acute effect of BAC on the cornea, we conducted studies by examining the alterations of cell proliferation and apoptosis.

**Figure 6:**
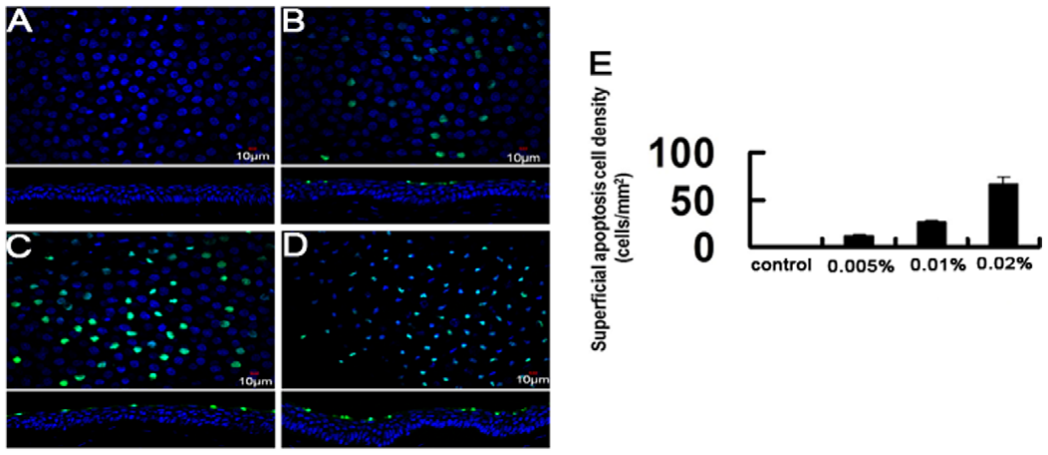
Acute effects of BAC on rabbit corneal epithelial cell apoptosis. Representative images for TUNEL assay (A–D). Upper panel shows representative images for TUNEL assay in the superficial epithelia. The lower panel shows an orthogonal view generated from cross-sectional studies. Mean superficial apoptotic cells were shown in (E). Data show mean ± SD of values from three eyes per group.

We detected the expression of Ki67 in the corneal epithelium following exposure to BAC. Ki67 protein, which is present during active phases of cell cycle (G1, S, G2 and mitosis), but is absent from resting cell (G0), has been considered to be an excellent marker for determining the growth fraction of a given cell population [30]. Immunofluorescence labeling of control corneal tissues with Ki67 antibody showed that Ki67 positive cells were located in the basal layer (12.0±5.3 cells/mm^2^), but not in the superficial layer. BAC treatment did not induced significant change of the number of Ki67 positive cells in the basal layer (Fig. 6E).

**Figure 7:**
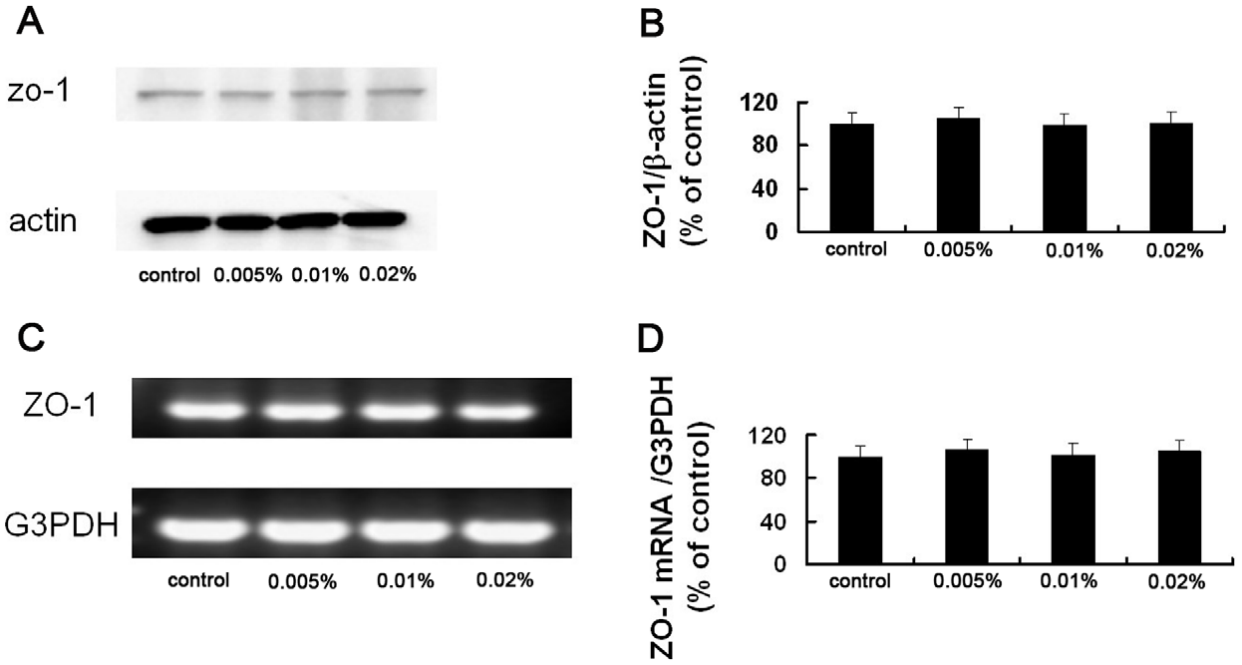
Acute effect of BAC on the expression of ZO-1 mRNA and protein in the rabbit corneal epithelium. (A) Corneal epithelial cells isolated from a control eye or from eyes treated with 0.005%, 0.01% or 0.02% BAC were subjected to western blot analysis with antibodies to ZO-1 or β actin (loading control). (B) Qutantitative analysis of ZO-1 band intensity in bolts similar to those shown in (A). Data were normalized by the corresponding β actin band intensity and mean ± SE of values from three eyes per group. (C) Corneal epithelial cells isolated from a control eye or from eyes treated with 0.005%, 0.01% or 0.02% BAC were subjected to RT-PCR analysis of ZO-1 mRNA. (D) Quantitative analysis of ZO-1 band intensity in gel similar to that shown in (A). Data were normalized by the corresponding G3PDH band intensity and are mean±SE of values from three eyes per group. Topical application of BAC had no significant effect on the amount of ZO-1 mRNA and protein (Dunnett test).

No or very rare TUNEL positively stained cells could be found in the control corneas (Fig. 7A). BAC concentrations from 0.005% to 0.02% significantly increased the number of TUNEL-positive cells in a dose-dependent manner with 12.3±2.6, 35.5±7.8, and 67±10.5 cells/mm^2^ after 0.005%, 0.01%, and 0.02% BAC treatments, respectively (Fig. 7B–E). Cross-sectional studies revealed that all TUNEL positively stained cells were located in the epithelial superficial layer (Fig. 7A–D).

## Discussion

BAC is the most frequently used preservative in multi-dose eye drops, and its side effects have been widely investigated [21], [31]. Previous study has suggested that toxicity of BAC on the ocular surface due to its slow turnover and its high ocular retention (up to 168 hours after administration of a single drop) [32]. In the present study, we utilized a clinically relevant model in rabbit to examine the *in vivo* acute effect of BAC on the localization and expression of ZO-1, a major component of tight junctions, in the corneal epithelium. We found that exposure to BAC quickly disrupted the localization of TJ-associated proteins in the rabbit corneal epithelium. We observed that BAC induces disruption of the corneal epithelial barrier function as assessed by corneal *in vivo* TER and CF uptake.

Fluorescein staining and rose Bengal staining are common methods for ocular surface evaluation. A recent study about dry eyes has demonstrated that fluorescein punctuate staining is the result of uptake caused by corneal superficial epithelial erosions [33]. Rose bengal has been found to stain corneal and conjunctival epithelial cells that are not adequately covered with tear film [34]. In this study, we found that exposure to BAC resulted in higher rose Bengal staining scores while no significant changes in BUT, Schirmer and corneal florescein scores. These findings are agreement with those studies reporting that in dry eye rose bengal staining is apparent earlier than fuorescein staining and BUT [34], [35].

We measured the *in vivo* TER after topical application of different concentrations of BAC to evaluate acute changes in corneal barrier function. Kusano et al. recently developed an *in vivo* corneal TER measurement system that can measure the barrier function of the intact fresh cornea in live rabbits [25]. They found that acute corneal barrier disruption occurs within only 5 seconds of instillation with 0.02% BAC. In another study, they evaluated acute corneal epithelial toxicity induced by BAC homologs with different alkyl chain lengths using this in vivo electrophysiological method [36]. They showed that acute corneal epithelial toxicity induced by BAC homologs depends on the alkyl chain length. Our study extends these and confirms that exposure to BAC can quickly induce the disruption of tight junctions between superficial cells in the rabbit corneal epithelium *in vivo*.

An important role of the corneal epithelium is to provide a functional barrier between the external and internal ocular environments. Tight junctions contribute to the formation of this barrier by controlling the extent and selectivity of diffusion along the paracellular pathway. Previous studies have proven that exposure to BAC disrupts corneal epithelial barrier function *in vitro* [37], [38]. Recently, we found that topical application of high concentration of BAC can induce the disruption of tight junctions between the rabbit corneal endothelial cells *in vivo* [39]. ZO-1 plays an important role in maintaining the barrier function and has been considered a marker of the tight junctions in the corneal epithelium [15], [16]. In this study, we found that control eyes exhibited a continuous linear pattern of tight junction proteins staining at cell-cell boundaries at the surface of the rabbit corneal epithelium *in vivo*. In the eyes treated with BAC, ZO-1 and ZO-2 immunoreactivity was patchy and discontinuous, suggestive of the disruption of tight junction. In normal corneal epithelium, ZO-1 is contiguous at cell-cell borders, and its dislocation is a marked indication of the loss of barrier integrity [38]. We noted that BAC induced dispersion of ZO-1 and ZO-2 from the interfaces of neighboring corneal superficial epithelial cells without affecting the localization of occludin and claudin-1. The mechanism by which BAC can quickly change the localization of MAGUK proteins without affecting that of occludin and claudin-1 in rabbit corneal epithelial cells remained to be determined. We also noted that there were no significant difference between control and BAC-treated corneal epithelia in amounts of ZO-1 mRNA and protein. These findings are consistent with the study of Kimura et al. in simian virus 40-transformed human corneal epithelial (HCE) cells, which also showed that the disappearance of ZO-1 and occludin from the interfaces of adjacent HCE cells induced by IL-1β was not accompanied by downregulation of the expression of these proteins [39]. Taken together, our results have demonstrated that short- and repeated exposure to BAC can quickly disrupt the barrier integrity of the cornea as a consequence of dispersion of MAGUK proteins from their normal locus at the superficial epithelial layer, even ZO-1 mRNA and protein expression in normal level.

*In vivo* confocal microscopy is a method widely used to observe morphologic changes of the cornea at the cellular level. We previously found that rabbit eyes treated with BAC showed significant change in the size of the corneal superficial epithelial cells compared with control eyes on examination by in vivo confocal microscopy [40]. We have now confirmed this finding. Furthermore, in vivo confocal microscopy revealed blur or loss of cell borders at the surface of the corneal epithelium in eyes exposed to BAC. This change was associated with BAC-induced disruption of the pattern of MAGUK proteins staining at the epithelial surface. Our results thus suggest that *in vivo* confocal microscopy may serve as a noninvasive and effective tool for evaluation of the barrier integrity of corneal epithelium.

We evaluated cell proliferation and apoptosis of rabbit corneal epithelium after BAC treatment. The results showed that short exposure to BAC did not induced significant change of the number of Ki67 positive cells in the corneal epithelium. In cultivation of chang conjunctival cells, BAC has been shown to induce apoptosis at low concentrations and necrosis at high concentrations [41]. In 3D-reconstituted corneal epithelium, apoptotic cells were only present in the superficial cell layer following exposure to low concentration (0.001%) of BAC [42]. Increasing BAC concentrations induced apoptosis in an increasing number of cellular layers, from the superficial to the basal layer. Our results support these observations, and show that exposure to BAC can quickly induce a significant dose-dependent increase of corneal epithelial cell apoptosis.
